# The Expression Pattern of the Pre-B Cell Receptor Components Correlates with Cellular Stage and Clinical Outcome in Acute Lymphoblastic Leukemia

**DOI:** 10.1371/journal.pone.0162638

**Published:** 2016-09-09

**Authors:** Dongfeng Chen, Junxiong Zheng, Natalija Gerasimcik, Kristina Lagerstedt, Helene Sjögren, Jonas Abrahamsson, Linda Fogelstrand, Inga-Lill Mårtensson

**Affiliations:** 1 Department of Rheumatology and Inflammation Research, Institute of Medicine, University of Gothenburg, Gothenburg, Sweden; 2 Department of Clinical Chemistry, Sahlgrenska University Hospital, Gothenburg, Sweden; 3 Queen Silvia Children’s Hospital, Gothenburg, Sweden; 4 Department of Clinical Chemistry and Transfusion Medicine, Institute of Biomedicine, University of Gothenburg, Gothenburg, Sweden; B.C. Cancer Agency, CANADA

## Abstract

Precursor-B cell receptor (pre-BCR) signaling represents a crucial checkpoint at the pre-B cell stage. Aberrant pre-BCR signaling is considered as a key factor for B-cell precursor acute lymphoblastic leukemia (BCP-ALL) development. BCP-ALL are believed to be arrested at the pre-BCR checkpoint independent of pre-BCR expression. However, the cellular stage at which BCP-ALL are arrested and whether this relates to expression of the pre-BCR components (*IGHM*, *IGLL1* and *VPREB1)* is still unclear. Here, we show differential protein expression and copy number variation (CNV) patterns of the pre-BCR components in pediatric BCP-ALL. Moreover, analyzing six BCP-ALL data sets (n = 733), we demonstrate that *TCF3-PBX1* ALL express high levels of *IGHM*, *IGLL1* and *VPREB1*, and are arrested at the pre-B stage. By contrast, *ETV6-RUNX1* ALL express low levels of *IGHM* or *VPREB1*, and are arrested at the pro-B stage. Irrespective of subtype, ALL with high levels of *IGHM*, *IGLL1* and *VPREB1* are arrested at the pre-B stage and correlate with good prognosis in high-risk pediatric BCP-ALL (n = 207). Our findings suggest that BCP-ALL are arrested at different cellular stages, which relates to the expression pattern of the pre-BCR components that could serve as prognostic markers for high-risk pediatric BCP-ALL patients.

## Introduction

B-cell precursor acute lymphoblastic leukemia (BCP-ALL) is one of the most common cancers in children, and accounts for 70% of leukemia in the 0 to 14 year age group. Based on cytogenetic aberrations, BCP-ALL can be divided into subtypes, e.g. t(12;21)(p13;q22) *ETV6-RUNX1*, t(1;19)(q23;p13) *TCF3-PBX1*, t(9;22)(q34;q11) *BCR-ABL1*, rearrangement of *MLL* (*KMT2A*) and high hyperdiploidy (≥50 chromosomes). Around 25% of patients do not fall within any of these categories and are often referred to as ‘other’. Correct subtype classification is important for risk assessment of patients with BCP-ALL. For example, *MLL*-rearrangement is associated with poor and *ETV6–RUNX1* with good prognosis. Most of the recurrent chromosomal aberrations are not sufficient for leukemia initiation by themselves, thus requiring the presence of additional cooperating lesions, e.g. in transcription factors that are crucial for B-cell development [1–3].

The early stages of B-cell developmental can be divided into progenitor B (pro-B), precursor B (pre-B) and immature B cells that can be defined by the expression of intracellular and/or cell surface markers in combination with the recombination status and expression of antibody (immunoglobulin, Ig) heavy (H) and light (L) chain genes [4]. In pre-B cells, Ig μH chains together with the invariant surrogate light (SL) chain, encoded by the *VPREB1* and *IGLL1* genes, form a pre-B cell receptor (pre-BCR), whereas in immature B cells μH chains and *bona fide* L chains form a BCR, i.e. a membrane bound antibody [5, 6]. Signaling from the pre-BCR (and BCR) is transmitted through the Igα/β (CD79A, CD79B) trans-membrane molecules. Pre-BCR expression constitutes a checkpoint that ensures productive V_H_DJ_H_ recombination and μH chain expression as cells develop from the pro- to pre-B stage [7]. Pre-BCR-mediated signaling induces Ig allelic exclusion, down-regulation of the recombination machinery, proliferation, survival, and subsequently cell cycle arrest and differentiation [8–10].

Tonic pre-BCR signaling is necessary for leukemia cell survival in BCP-ALL (mainly *TCF3-PBX1*) expressing a pre-BCR [11, 12]. By contrast, in *BCR-ABL1* ALL pre-BCR expression is down-regulated and the need for pre-BCR signaling is abolished due to the kinase bypassing this receptor by ‘hijacking’ some of its downstream signaling [13]. These results together with other studies have led to a model in which BCP-ALL are arrested at the pre-BCR checkpoint due to one of the following mechanisms [14, 15]: (i) expressing a pre-BCR that mediates signals supporting survival and proliferation but inhibiting differentiation, (ii) bypassing the pre-BCR checkpoint, or (iii) blocking/down-regulating pre-BCR expression, and through alternative mechanisms activating proliferation and survival. To gain further insight into the importance of the pre-BCR in BCP-ALL, we have investigated the expression patterns of the pre-BCR components in childhood BCP-ALL in relation to genetic subtype, differentiation stage and clinical outcome.

## Materials and Methods

### Patient samples

The cohort at the Sahlgrenska University Hospital included 123 children diagnosed with BCP-ALL from 2007 to 2015. The diagnosis was based on morphological review of bone marrow smears, immunophenotyping, cytogenetics and molecular genetics according to the WHO 2008 criteria [16]. All studies using human patient samples are approved by the Regional Ethical Review Board in Gothenburg (415–12), and the patients and/or their guardians provided written informed consent. Experiments were done in compliance with ethical guidelines and without any clinical interventions.

### Flow cytometry

Bone marrow samples from 24 patients were harvested and analyzed at the time of first diagnosis. The BCP-ALL bone marrow samples were stained with antibodies recognizing VPREB1 (HSL96-PE) and IGLL1 (HSL11-PE) in parallel with diagnostic antibodies e.g. CD19-PeCy7, CD45-APC-H7, CD10-APC, CD34-PerCP, and intracellular IGHM-FITC, TdT-FITC, CD79A-PE. For each staining, 10,000 cells were acquired on a FACSCanto II (BD, USA) and analyzed using FlowJo software (FlowJo, LLC, USA).

### Copy number variation analysis

High resolution DNA copy number variation (CNV) analysis was performed on all patients at the time of diagnosis using the Affymetrix platform, ie the GeneChip^®^ Human Mapping Affymetrix 250K (Affymetrix, Inc., Santa Clara, CA) from 2007 to 2010, the Genome wide Human SNP 6.0 during 2010 and the CytoscanHD^®^ array from 2011. These three platforms contain 500 000, 906 600 and 750 000 single nucleotide polymorphisms (SNPs), respectively. The raw CEL files were analyzed for DNA CNV using Nexus Copy Number software (BioDiscovery, USA).

### Gene expression microarray data

Gene expression microarray data of pediatric BCP-ALL and healthy B cells were gathered from published studies [17–24]. The healthy B-cell (GSE45460) and leukemia data sets (GSE12995, GSE26281, GSE17703, GSE13425, GSE47051 and GSE11877) were downloaded from NCBI GEO dataset. Data set Blood 2003 was collected from a previous BCP-ALL study [17].

### Gene Set Enrichment Analysis

To assess similarity of molecular signatures between different BCP-ALL subtypes or clusters and different normal B cell developmental stages, gene set enrichment analysis (GSEA) was performed using Qlucore Omics Explorer 3.2 (Qlucore AB, Lund, Sweden).

### Analysis of survival and minimal residual disease data

The clinical data from the 207 high-risk patients in data set GES11877 were collected from a previous study [13]. The description of these high-risk patients can be found at: http://www.ped-onc.org/diseases/ALLtrials/9906.html. Overall survival was estimated by the Kaplan-Meier method (Prism 6.0). Survival differences were assessed with the log-rank test. Overall survival time was defined as time span from the date of diagnosis until death or the data of last follow-up contact. Comparison of minimal residual disease day 29 (MRD 29) data was determined with Fisher's exact test.

## Results

### Differential protein expression pattern of the pre-BCR components in BCP-ALL

**Fig 1:**
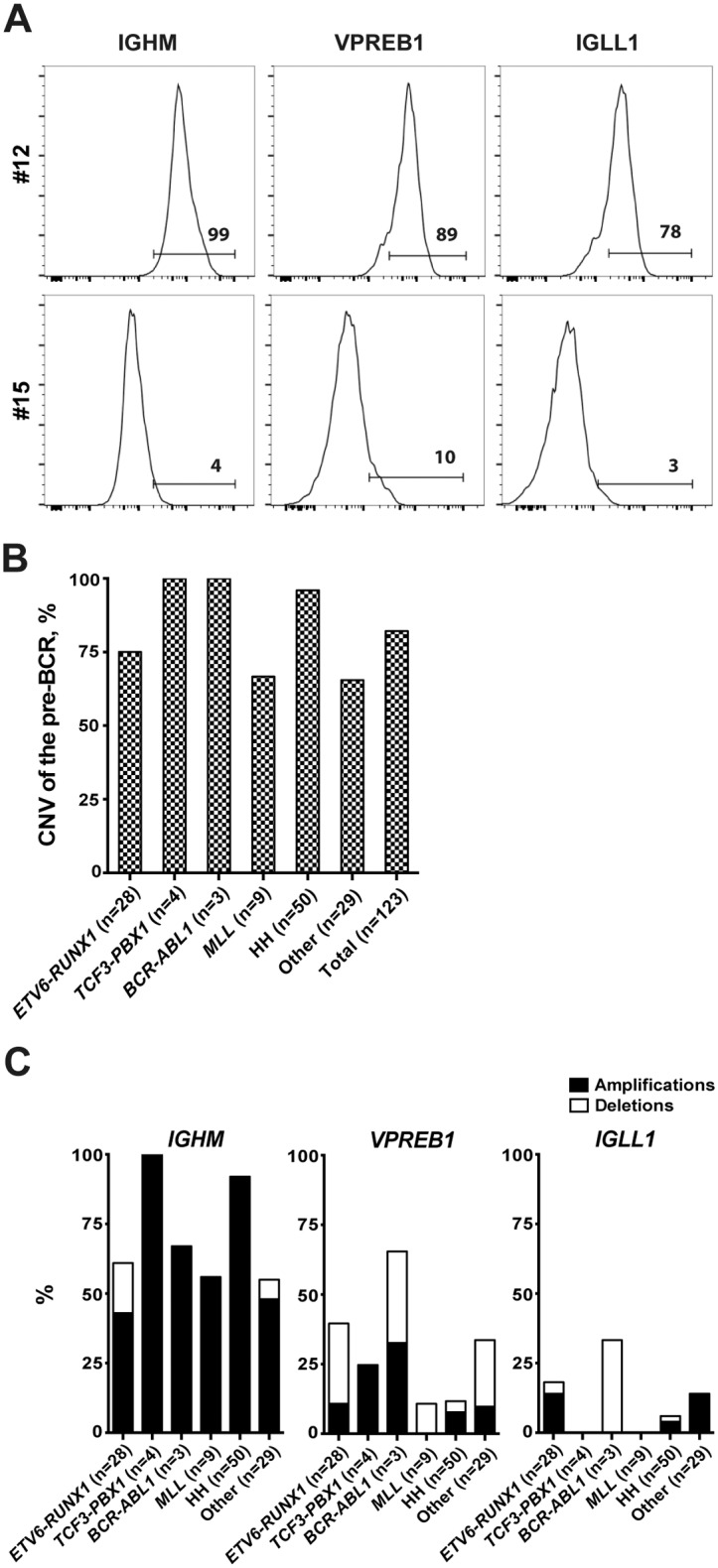
The pre-BCR components show differential protein expression and copy number variation patterns in BCP-ALL patients. (A) Histograms show the differential expression patterns of IGHM, VPREB1 and IGLL1 in CD19^+^-blasts from two patients (#12 and #15). Bone marrow samples were stained with antibodies against μ heavy chain (IGHM), CD179a (VPREB1) and CD179b (IGLL1), and then analyzed by flow cytometry. (B) Copy number variation (CNV) analysis shows the genetic aberration frequencies of the pre-BCR in 123 patients. The pre-BCR is defined as aberrant when one or more of the pre-BCR components showed CNVs. (C) CNV analyses show the percentage of amplifications and/or deletions of *IGHM*, *VPREB1* and *IGLL1*. HH, High Hyperdiploid.

To determine the protein expression pattern of the pre-BCR components, a cohort of 24 pediatric BCP-ALL patient samples were analyzed by flow cytometry (Fig 1A and S1 Table). As expected, all samples were CD19 and CD79A positive. Moreover, all samples except one stained positive for CD10 and TdT. Around half of the samples, including the only *TCF3-PBX1* ALL, were positive for intracellular μH chain (IGHM), whereas four of the five *ETV6-RUNX1* ALL were negative. A large majority of samples were positive for intracellular VPREB1 and/or IGLL1 but the proportion of positive cells varied (S1 Table). Thus, a differential protein expression pattern was observed for the pre-BCR components.

### Copy number variation of the pre-BCR components in BCP-ALL

To determine whether the expression pattern of the pre-BCR components was associated with gene copy number variation (CNV), SNP array data from 123 pediatric BCP-ALL was analyzed (including the above 24). In a majority of the cases, at least one of the pre-BCR components showed either deletion or amplification (Fig 1B). As expected, *IGHM* and *CD79B*, located on chromosomes 14 and 17, were frequently amplified in hyperdiploid, presumably due to frequent gain of these two chromosomes (Fig 1C and S2 Table). Excluding this subtype, most CNVs were observed in *IGHM* (61%), *VPREB1* (34%) and *IGLL1* (14%) whereas CNVs were infrequent in *CD79A* (1%) and *CD79B* (5%). For *IGHM*, amplifications were more frequent than deletions whereas in *VPREB1* deletions, mostly mono-allelic, were more frequent. Among the 24 samples in which both flow cytometry and CNV were performed, there were no associations between the presence of amplification or deletion and the protein expression for any of the pre-BCR components (p>0.05, S1 Table). Thus, a differential CNV pattern of the pre-BCR components was observed, which did not associate with their protein expression pattern.

### Distinct mRNA expression patterns of the pre-BCR components in BCP-ALL

**Fig 2:**
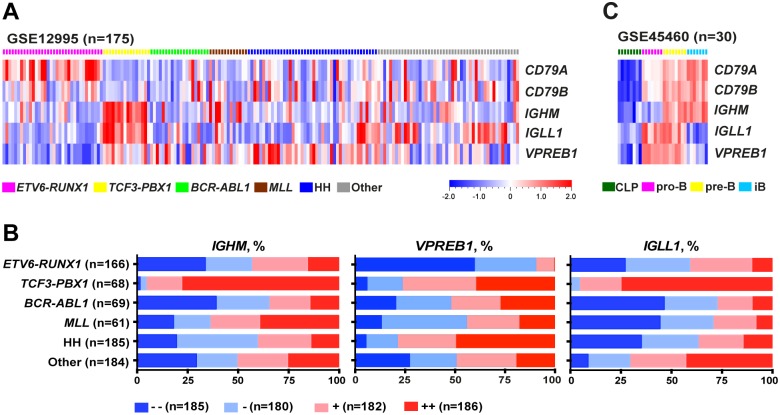
The pre-BCR components show distinct mRNA expression patterns in BCP-ALL and normal B cells. (A) Heat map shows the expression patterns of the pre-BCR components in childhood BCP-ALL (GSE12995). (B) The graphs show the expression patterns of *IGHM*, *VPREB1* and *IGLL1* in 733 BCP-ALL patient samples from six cohorts (GSE12995, Blood2003, GSE177031, GSE26281, GSE13425 and GSE47051). All patient samples were evenly divided into quartiles according to the expression level of *IGHM*, *VPREB1* or *IGLL1*. (C) Heat map shows the expression patterns of the pre-BCR components in a normal B-cell data set (GSE45460). HH, High Hyperdiploid; iB, immature B cells.

To determine whether the differential protein expression pattern of the pre-BCR components is also evident at the mRNA level, we analyzed six childhood BCP-ALL data sets with a total of 733 patients (Fig 2A and 2B, S1 Fig). For *ETV6-RUNX1* ALL (n = 166), the most striking observation was the low levels of at least one pre-BCR component, most often *VPREB1*, and high levels of *CD79A* in the majority of samples. By contrast, the vast majority of *TCF3-PBX1* ALL (n = 68) expressed high levels of *IGHM*, *VPREB1* and *IGLL1*, although the levels of *CD79A* and *CD79B* were low. Within the other subtypes, the most prominent features were low levels of *IGLL1* in *BCR-ABL1* (n = 69), *MLL* (n = 61) and hyperdiploid ALL (n = 185). Moreover, the levels of *VPREB1* and *IGLL1* were high in hyperdiploid and ‘other’ ALL (n = 184), respectively. Thus, the different subtypes of BCP-ALL display distinct gene expression patterns of the pre-BCR components: a majority of *TCF3-PBX1* ALL express high levels of *IGHM*, *VPREB1* and *IGLL1* whereas *ETV6-RUNX1* and the remaining ALL do not.

To compare the mRNA expression levels of the pre-BCR components in BCP-ALL with those in B-lineage cells, we analyzed a normal B-cell data set including also common lymphoid progenitors (CLPs). As expected, CLPs did not express any of the pre-BCR components, while pro-B cells expressed *VPREB1*, *IGLL1* and *CD79A/B*. In pre-B cells all pre-BCR components were expressed, while only *IGHM* and *CD79A/B* were detected in immature B cells (Fig 2C). Thus, the high levels of *IGHM*, *VPREB1* and *IGLL* observed in a majority of *TCF3-PBX1* ALL is a typical feature of pre-B cells, whereas the low level of at least one pre-BCR component found in most remaining ALL is atypical.

### *TCF3-PBX1* ALL and healthy pre-B cells display similar molecular signatures

**Fig 3:**
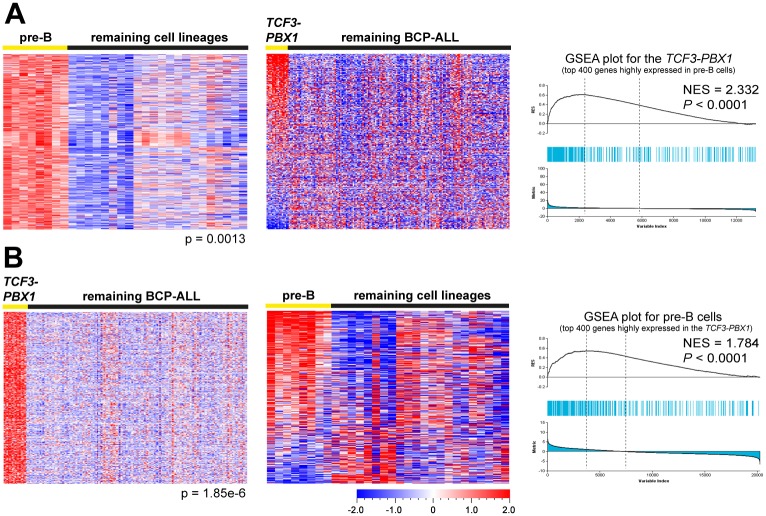
GSEA reveals molecular signature similarities between the *TCF3-PBX1* BCP-ALL and normal pre-B cells. (A) Genes highly expressed in pre-B cells are enriched in the *TCF3-PBX1*. Left: The top 400 genes highly expressed in healthy pre-B cells (pre-B signature) were identified using supervised comparison in data set GSE45460. Middle: Heat map of the pre-B signature in the *TCF3-PBX1* and the remaining ALL in data set GSE12995. Right: Enrichment plot shows the enrichment of pre-B signature in the *TCF3-PBX1*. The upper part of the Fig shows the enrichment score (NES), which is calculated by ranked ordered gene set and increasing the score when a gene is in the set and decreasing it when it is not. Each blue line represents a hit from the gene set. The more hits among the top up- or downregulated genes, the more likely a significant gene set enrichment score is gotten. The lower portion of the Fig shows the rank ordered genes for the *TCF3-PBX1* when compared to other ALL, with genes being highly expressed to the far left and downregulated genes to the right. (B) Genes highly expressed in the *TCF3-PBX1* are enriched in pre-B cells. Left: The top 400 genes highly expressed in *TCF3-PBX1* (*TCF3-PBX1* signature) were identified using supervised comparison. Middle: Heat map of the *TCF3-PBX1* signature in healthy pre-B cells. Right: Enrichment plot shows the enrichment of *TCF3-PBX1* signature in healthy pre-B cells.

Because a pre-BCR is expressed at the pre-B but not other cellular stages, the TCF3-PBX1 ALL might be arrested at the pre-B stage. To test this, we identified expression signatures consisting of the top 400 genes highly expressed at each of the four developmental stages in the healthy B-cell data set (S2 Fig and S1 File). Thereafter, using these signatures, we performed gene set enrichment analysis (GSEA) in each BCP-ALL data set to determine whether any of the ‘healthy’ signatures was enriched in a particular ALL subtype. The GSEA results showed that the pre-B signature was specifically enriched in the *TCF3-PBX1* but not other subtypes (Fig 3A, S3 Fig and S3 Table). Thereafter, we performed the reverse experiment, and identified the top 400 genes highly expressed in the *TCF3-PBX1* subtype in each BCP-ALL data set (S4A Fig and S2 File). The GSEA results showed that the *TCF3-PBX1* signatures were specifically enriched in pre-B cells (Fig 3B, S3 Fig and S3 Table). Thus, these results support the notion that a majority of *TCF3-PBX1* BCP-ALL not only express a pre-BCR but also are arrested at the pre-B stage.

### *ETV6-RUNX1* ALL and healthy pro-B cells show similar molecular signatures

**Fig 4:**
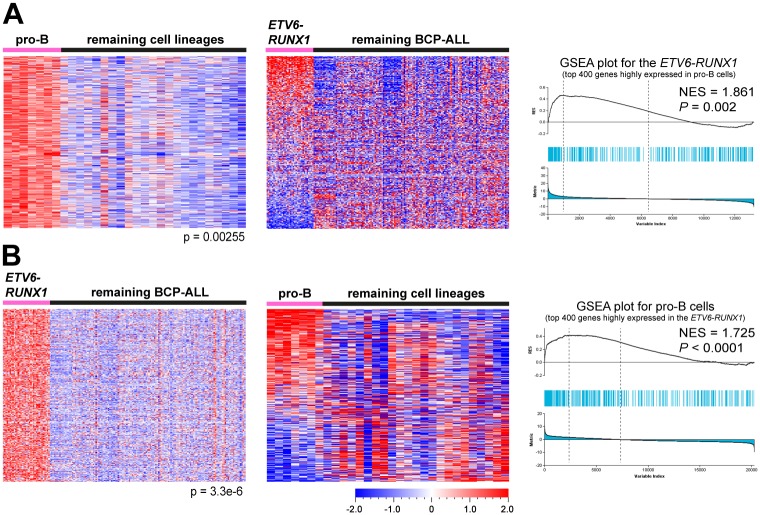
GSEA reveals molecular signature similarities between the *ETV6-RUNX1* BCP-ALL and normal pro-B cells. (A) Genes highly expressed in pro-B cells are enriched in the *ETV6-RUNX1*. Left: The top 400 genes highly expressed in pro-B cells (pro-B signature) were identified using supervised comparison in data set GSE45460. Middle: Heat map of the pro-B signature in the *ETV6-RUNX1* and the remaining ALL in data set GSE12995. Right: Enrichment plot show the enrichment of pro-B signature in the *ETV6-RUNX1* (B) Genes highly expressed in the *ETV6-RUNX1* are enriched in pro-B cells. Left: The top 400 genes highly expressed in the *ETV6-RUNX1* (*ETV6-RUNX1* signature) were identified using supervised comparison. Middle: Heat map of the *ETV6-RUNX1* signature in healthy pro-B cells. Right: Enrichment plot showing the enrichment of the *ETV6-RUNX1* signature in healthy pro-B cells.

In contrast to the *TCF3-PBX1* the other subtypes did not appear arrested at the pre-B cell stage, therefore, we hypothesized that these BCP-ALL subtypes might be arrested at other stages. To test this hypothesis we pursued the same strategy as above. We found that the pro-B signature was enriched in *ETV6-RUNX1* ALL in all four data sets (Fig 4A and S5 Fig). It was also enriched in *BCR-ABL1* ALL in three of the data sets but not with the same significance as in the *ETV6-RUNX1* subtype (S3 Table). Thereafter, we performed the reverse experiment, and identified the top 400 genes highly expressed in the *ETV6-RUNX1* and *BCR-ABL1* subtypes in the four data sets (S4B and S4C Fig, S3 and S4 Files). The GSEA results showed that the *ETV6-RUNX1* signatures were specifically enriched in pro-B cells, whereas the *BCR-ABL1* signatures were not enriched in any of the four ‘healthy’ stages (Fig 4B, S5 Fig and S3 Table). The remaining subtypes did not show molecular signatures similar to any of the B-cell developmental stages, or vice versa. Thus, these results suggest that a majority of *ETV6-RUNX1* BCP-ALL, of which a majority expressed low levels of at least one pre-BCR component, are arrested at the pro-B cell stage.

### *IGHM*^+^*VPREB1*^+^*IGLL1*^+^ high-risk ALL and healthy pre-B cells show similar molecular signatures

**Fig 5:**
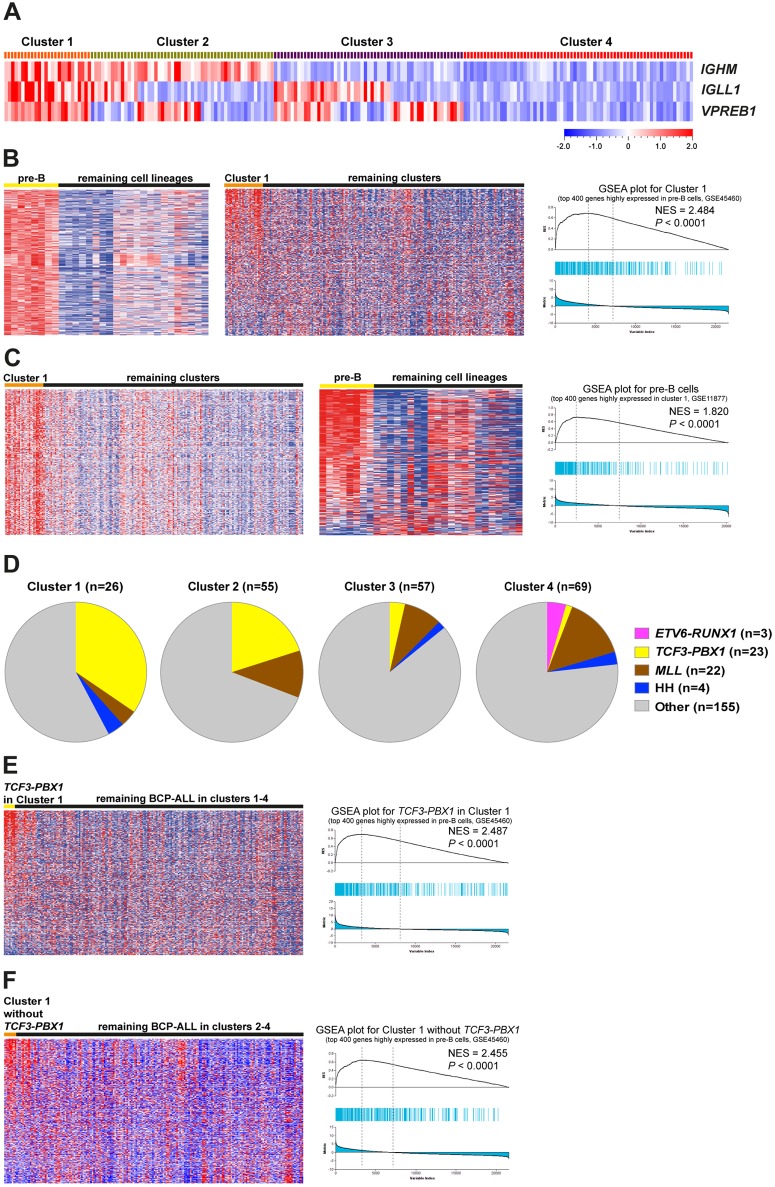
GSEA reveals molecular signature similarities between the *IGHM*^+^*IGLL1*^+^*VPREB1*^+^ BCP-ALL and normal pre-B cells. (A) The BCP-ALL in data set GSE11877, including 207 high-risk patient samples, were classified into four clusters according the expression levels of *IGHM*, *IGLL1* and *VPREB1*: *IGHM+IGLL1+VPREB1+* (Cluster 1), *IGHM+IGLL1+/-VPREB1+/-* (not including *IGHM+IGLL1+VPREB1+*) (Cluster 2), *IGHM-IGLL1+/-VPREB1+/-* (not including *IGHM-IGLL1-VPREB1-*) (Cluster 3) and *IGHM-IGLL1-VPREB1-* (Cluster 4). (B) Genes highly expressed in pre-B cells are enriched in Cluster 1. Left: The pre-B signature was identified using supervised comparison in data set GSE45460. Middle: Heat map of the pre-B signature in Cluster 1 and the remaining ALL. Right: Enrichment plot shows the enrichment of pre-B signature in the Cluster 1. (C) Genes highly expressed in Cluster 1 are enriched in pre-B cells. Left: The top 400 genes highly expressed in Cluster 1 (Cluster 1 signature) were identified using supervised comparison. Middle: Heat map of the Cluster 1 signature in healthy pre-B cells. Right: Enrichment plot shows the enrichment of Cluster 1 signature in healthy pre-B cells. (D) Pie-chart shows the distribution of genetic subtypes in clusters 1–4. (E) Genes highly expressed in pre-B cells are enriched in the *TCF3-PBX1* ALL from Cluster 1 but not that from other clusters. Left: Heat map of the pre-B signature in the *TCF3-PBX1* ALL from Cluster1. Right: Enrichment plot shows the enrichment of pre-B signature in the *TCF3-PBX1* ALL from Cluster 1. (F) Genes highly expressed in pre-B cells are enriched in Cluster 1 without *TCF3-PBX1*. Left: Heat map of the pre-B signature in the Cluster1 without *TCF3-PBX1*. Right: Enrichment plot shows the enrichment of pre-B signature in Cluster 1 without the *TCF3-PBX1* ALL.

To determine whether the expression pattern of the pre-BCR components correlate with clinical outcomes, we investigated a public data set including 207 high-risk pediatric BCP-ALL patient samples. First, we classified the samples into four clusters according to the expression levels of *IGHM*, *IGLL1* and *VPREB1* (Fig 5A). In Cluster 1 (*IGHM*^+^*IGLL1*^+^*VPREB1*^+^) all three genes were highly expressed, in Cluster 2 (*IGHM*^+^*IGLL1*^*+/-*^*VPREB1*^*+/-*^) *IGHM* and either *IGLL1* or *VPREB1* but not both were high, in Cluster 3 (*IGHM*^-^*IGLL1*^*+/-*^*VPREB1*^*+/-*^) *IGHM* was low whereas *IGLL1* and/or *VPREB1* were high, and in Cluster 4 (*IGHM*^-^*IGLL1*^-^*VPREB1*^-^) all three genes were lowly expressed. Then the GSEA was performed to determine whether any of the clusters displayed a molecular signature similar to any of B-cell developmental stages. The results showed that the pre-B signature was specifically enriched in Cluster 1, and also vice versa (Fig 5B and 5C). In addition, we determined whether a particular subtype was enriched in any of Cluster 1–4, and found that most of the *TCF3-PBX1* ALL were in Cluster 1 and 2 (Fig 5D). To assess whether the *TCF3-PBX1* in these two clusters showed a pre-B signature, GSEA was performed. The results revealed a pre-B signature in the *TCF3-PBX1* ALL in Cluster 1 (Fig 5E), but not in Cluster 2 (p<0.0001 versus p = 0.105). Moreover, after excluding the *TCF3-PBX1* samples, the ALL in Cluster 1 still showed a pre-B signature (Fig 5F), and hence the pre-B signature in Cluster 1 was not skewed by the presence of the *TCF3-PBX1* samples. We were unable to define consistent signatures in the remaining clusters. These results suggest that irrespective of genetic subtype *IGHM*^+^*VPREB1*^+^*IGLL1*^+^ BCP-ALL are arrested at the pre-B cell stage.

### BCP-ALL in Cluster 1–4 show different clinical outcomes

**Fig 6:**
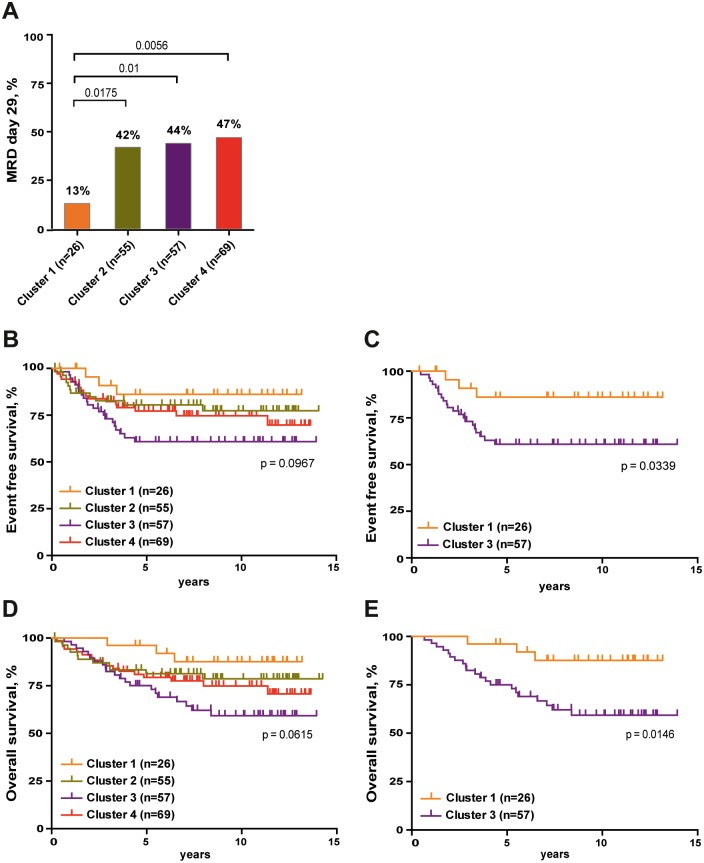
The expression pattern of pre-BCR components associates with the clinical outcomes in high-risk patient group. (A) The percentage of MRD 29 positive patients was compared among clusters 1–4 using Fisher's exact test. MRD 29: minimal residual disease at day 29. (B-E) The Kaplan-Meier Log rank Survival analysis was performed to compare event free survival (B and C) and overall survival (D and E) of the 207 high-risk patients. Survival probabilities between patients within different clusters (1–4) are shown.

To determine whether BCP-ALL in the four clusters showed different clinical outcomes, the clinical data from the 207 high-risk pediatric BCP-ALL patients was analyzed. Initial treatment response, which is correlated to outcome, was evaluated by minimal residual disease (MRD) 29 days after beginning of treatment. Analyzing the MRD data, we found that a lower proportion of patients in Cluster 1 were MRD positive on day 29 compared to those in the other three clusters, 13% versus 42–47% (Fig 6A). We also found that patients in Cluster 1 had longest event free survival and survived significantly longer than those in Cluster 3 with the shortest event free and overall survival time (Fig 6B–6E). Thus, the expression patterns of the pre-BCR components correlate with early treatment response, relapse and survival, where pre-BCR expression appears to signify better prognosis, at least in high-risk patients.

## Discussion

During early B-cell development expression of a pre-BCR serves as a quality checkpoint to ensure that pro-B cells that progress to the pre-B stage express a functional antibody μH chain. Here we find that a majority of BCP-ALL expresses low levels of at least one pre-BCR component, which is supported by previous work [11, 25]. We find distinct patterns of the pre-BCR components were consistently observed in *ETV6-RUNX1* and *TCF3-PBX1*. Most *ETV6-RUNX1* expressed low *IGHM* mRNA levels, possibly lacking protein expression as observed in our flow cytometry analysis. However, it is unclear whether a lack of IGHM protein is due to the recombination status of the IgH locus, *IGHM* genetic alterations or transcriptional silencing. In addition, most *ETV6-RUNX1* ALL expressed low mRNA levels of *VPREB1* and/or *IGLL1*. One possible explanation is that the *ETV6-RUNX1* fusion gene down-regulates *VPREB1/IGLL1* expression [26], another is the combination of the fusion gene with mono-allelic deletions of *VPREB1* and/or *IGLL1*. The reduced levels of *VPREB1*, *IGLL1* and/or *IGHM* could also be as a result of genetic aberrations in transcription factors [1, 2], e.g. *E2A*, *EBF* and *IKZF1* frequently found in BCP-ALLs, as these factors regulate their transcription and are crucial for B cell development [27–30].

Our results suggest that *ETV6-RUNX1* ALL are arrested at the pro-B cell stage. In humans, as well as in mouse models, mutations in, or absence of *IGHM*, *VPREB1*, *IGLL1* or *CD79A/B* causes either an arrest or a severe impairment at the pro-B cell stage [6, 7]. Although heterozygosity does not appear to affect B-cell development, a gradual impairment is observed when *VPREB1* mRNA levels are successively reduced below 50% [31]. These results could serve as explanations to the arrest of *ETV6-RUNX1* ALL at the pro-B stage. Moreover, leukemic cells arrested at the pro-B stage would not have to bypass the pre-BCR checkpoint but rather rely on survival signals typical at this stage.

By contrast, we find that *TCF3-PBX1* ALL express high levels of *IGHM*, *VPREB1* and *IGLL*, and are most likely pre-BCR^+^ [11]. Our results argue that *TCF3-PBX1* ALL are arrested at the pre-B cell stage, which would be consistent with pre-BCR expression. In a high-risk BCP-ALL data set the samples in Cluster 1 (*IGHM*^+^*VPREB1*^+^*IGLL1*^+^*)* are also arrested at the pre-B stage. That pre-BCR^+^ leukemic cells are arrested at the pre-B stage is supported by animal models, where aberrant signaling downstream of the pre-BCR gives rise to spontaneous leukemia [32]. Considering the proposed model of BCP-ALL development [14, 15], our data support the notion that pre-BCR^+^ ALL are arrested at the pre-B cell stage, e.g. most *TCF3-PBX1*. The model also suggests that some ALL bypass the pre-BCR checkpoint and/or downregulate pre-BCR expression, inferring that these ALL are still arrested at the pre-B stage. However, our results do not support that pre-BCR^-^ ALL are arrested at the pre-B cell stage.

Although gene expression profiling has uncovered unique signatures correlating with BCP-ALL genetic subtypes [33, 34], it is unclear whether these signatures relate to defined stages of normal B-cell development. Also, BCP-ALL can be classified as pro-B, common or pre-B ALL based on the expression of markers such as CD19, CD10 and intracellular IGHM, TdT (*DNTT*) and Igα (*CD79A*) determined by flow cytometry [35]. However, these markers does not necessarily define the developmental stage where the leukemia cells are arrested, as their expression might be de-regulated, which is evident in that almost all BCP-ALL express TdT that is only expressed in healthy pro-B cells. Nevertheless, here we find that the *ETV6-RUNX1* and *TCF3-PBX1* signatures are enriched in pro-B and pre-B cells, respectively, and vice versa. A majority of the genes in the healthy pro- or pre-B signatures are involved in cellular processes such as cell cycle progression, cell survival and proliferation rather than characteristic of B-lineage cells. There are a few exceptions though, e.g. TdT (*DNTT*) that is involved in VDJ recombination, the tyrosine kinase SYK that is involved in signaling downstream of the pre-BCR, and LYN that is involved in signaling downstream of the BCR are found in the pro-B, pre-B and immature B signature, respectively. Furthermore, consistent with previous studies [33, 36], in the *ETV6-RUNX1* signature we found KCNN1 and PTP4A3 that are involved in signaling, and in the *TCF3-PBX1* signature we found MAD1L that is involved in mitosis, as well as MERTK, a receptor tyrosine kinase.

Previous work has pointed to the importance of BCL6 downstream of the pre-BCR, where the signaling molecules SYK and BTK would regulate its expression [11]. Consistent with this we find all three genes being expressed in pre-BCR^+^ pre-B cells as well as in most pre-BCR^+^ *TCF3-PBX1* (S6 Fig). The levels of these same molecules were low in most pre-BCR^-^ *ETV6-RUNX1*, which instead expressed *STAT5B* and *CCND2*. However, we find that normal pre-BCR^-^ pro-B cells, in addition to *STAT5B* and *CCND2*, expressed *BCL6*. This indicates that this transcription factor is not only under control of the pre-BCR—in pre-B cells—but also downstream of other receptors—in pro-B cells—despite the expression of *STAT5B*.

In summary, our data demonstrate that the expression pattern of the pre-BCR components in BCP-ALL correlates with different B cell developmental stages and that high mRNA levels of the pre-BCR components correlate with good prognosis in high-risk patients.

## Supporting Information

S1 Fig The pre-BCR components show distinct mRNA expression patterns in childhood BCP-ALL patients. (A) Heat map shows the expression patterns of the pre-BCR components in three childhood BCP-ALL cohorts (GSE26281, Blood2003, and GSE177031). (B) Meta-analysis shows the expression patterns of *CD79A* and *CD79B* in 733 BCP-ALL patients from six cohorts (GSE12995, Blood2003, GSE177031, GSE26281, GSE13425 and GSE47051). All BCP-ALL patient samples are evenly classified into four clusters according to the expression level of *CD79A* or *CD79B*.
(TIF)

S2 Fig Heat map shows the molecular signatures of the cells in different normal B developmental stages. The top 400 genes highly expressed in different developmental stages (CLP, pro-B, pre-B and iB signatures) were identified using supervised comparison in the data set including samples from healthy donors (GSE45460). iB, immature B.
(TIF)

S3 Fig GSEA reveals molecular signature similarities between the *TCF3-PBX1* BCP-ALL and normal pre-B cells. (**A-C**) Upper: Heat map and enrichment plots show the pre-B signature in the *TCF3-PBX1* and the remaining BCP-ALL in the indicated data sets. Lower: Heat map and enrichment plots show the *TCF3-PBX1* signature in pre-B cells and the remaining developmental stages.
(TIF)

S4 Fig Heat map shows the molecular signatures of different BCP-ALL subtypes. (A) The top 400 genes highly expressed in the *TCF3-PBX1* BCP-ALL (*TCF3-PBX1* signature) are identified using supervised comparison. (B) The top 400 genes highly expressed in the *ETV6-RUNX1* BCP-ALL (*ETV6-RUNX1* signature) are identified using supervised comparison. (C) The top 400 genes highly expressed in the BCR-ABL1 BCP-ALL (*BCR-ABL1* signature) are identified using supervised comparison.
(TIF)

S5 Fig GSEA reveals molecular signature similarities between the *ETV6-RUNX1* BCP-ALL and normal pro-B cells. (A-C) Upper: Heat map and enrichment plots show the pro-B signature in the *ETV6-RUNX1* and the remaining BCP-ALL in the indicated data sets. Lower: Heat map and enrichment plots show the *ETV6-RUNX1* signature in pro-B cells and the remaining developmental stages.
(TIF)

S6 Fig Pre-BCR and *BCL6* expression correlates in BCP-ALL but not normal B cells. (A and B) Heat maps show the expression patterns of indicated molecules in normal B-cell (GSE45460) and in childhood BCP-ALL (GSE12995) data sets. (A) *IGHM*, *BCL6*, *SYK* and *BTK* expression in pre-B cells and *TCF3-PBX1* (boxed). (B) *IGHM*, *BCL6*, *STAT5B* and *CCND2* expression in pro-B cells and *ETV6-RUNX1* (boxed). iB, immature B cells.
(TIF)

S1 File Top 400 genes highly expressed in different healthy B cell developmental stages. (XLSX)

S2 File Top 400 genes highly expressed in TCF3-PBX1 subtypes in different data sets. (XLSX)

S3 File Top 400 genes highly expressed in ETV6-RUNX1 subtypes in different data sets. (XLSX)

S4 File Top 400 genes highly expressed in BCR-ABL1 subtypes in different data sets. (XLSX)

S1 Table Summary of Flow Cytometry and Copy Number Variation data from the cohort at the Sahlgrenska University Hospital. (DOCX)

S2 Table Copy Number Variation data of the pre-BCR components from the cohort at the Sahlgrenska University Hospital. (DOCX)

S3 Table Similar molecular signatures between BCP-ALL and healthy B cells. The top 400 genes highly expressed at each of the four B cell developmental stages in data set DS5 were projected into data sets DS1-4 (Healthy -> Leukemia) and vice versa (Leukemia -> Healthy). Then gene set enrichment analyses were performed to determine the similarity of molecular signatures between BCP-ALL and normal B cells. Only significant p-values (>0.05) are shown.
(DOCX)
