# Supplementary figures and images for: The Expression Pattern of the Pre-B Cell Receptor Components Correlates with Cellular Stage and Clinical Outcome in Acute Lymphoblastic Leukemia
Source: PLoS One. 2016 Sep 9;11(9):e0162638. doi: 10.1371/journal.pone.0162638 (PMC5017602; doi:10.1371/journal.pone.0162638)

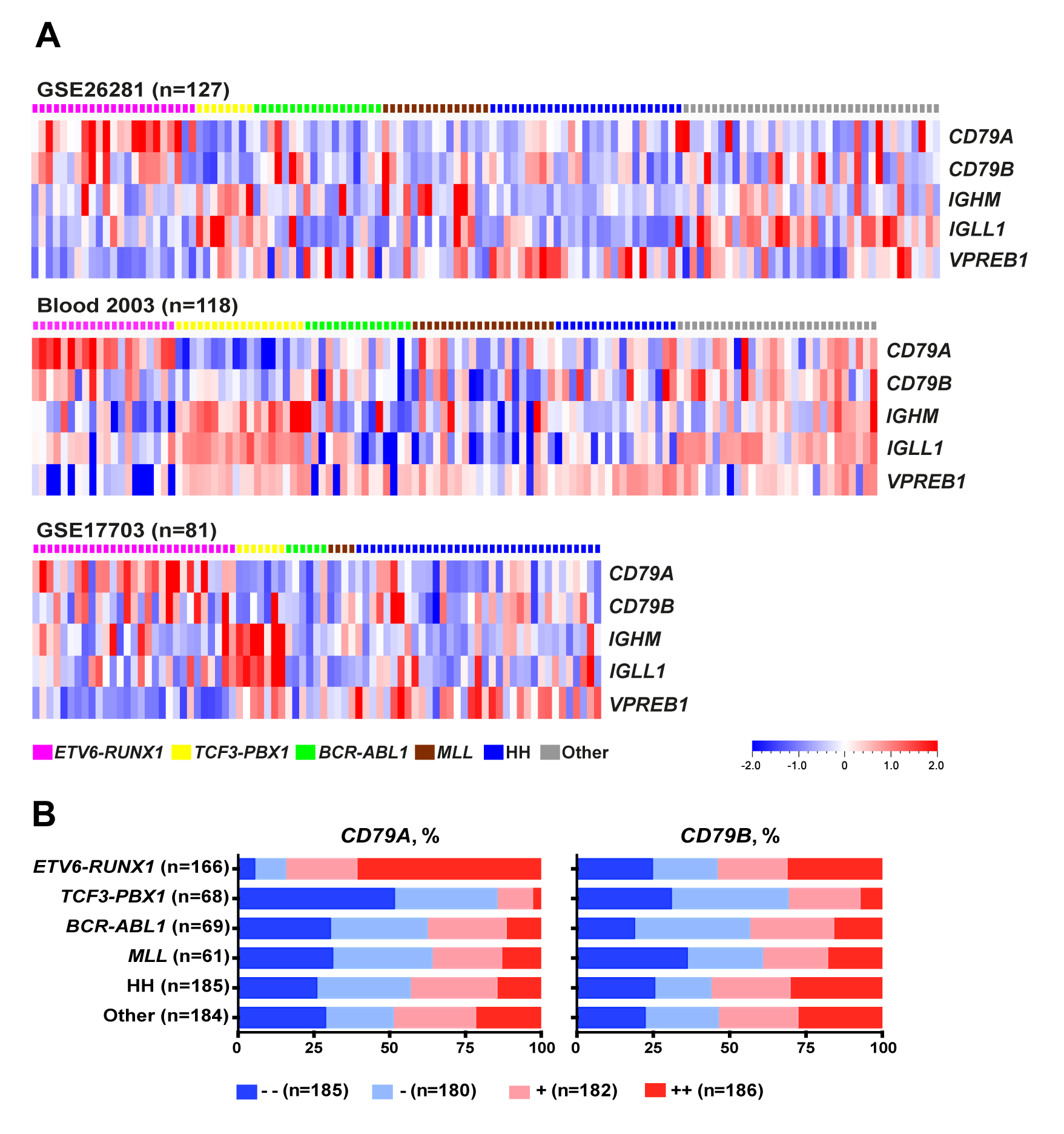

Supplement: S1 Fig — (A) Heat map shows the expression patterns of the pre-BCR components in three childhood BCP-ALL cohorts (GSE26281, Blood2003, and GSE177031). (B) Meta-analysis shows the expression patterns of CD79A and CD79B in 733 BCP-ALL patients from six cohorts (GSE12995, Blood2003, GSE177031, GSE26281, GSE13425 and GSE47051). All BCP-ALL patient samples are evenly classified into four clusters according to the expression level of CD79A or CD79B. (TIF) [file pone.0162638.s001.tif]

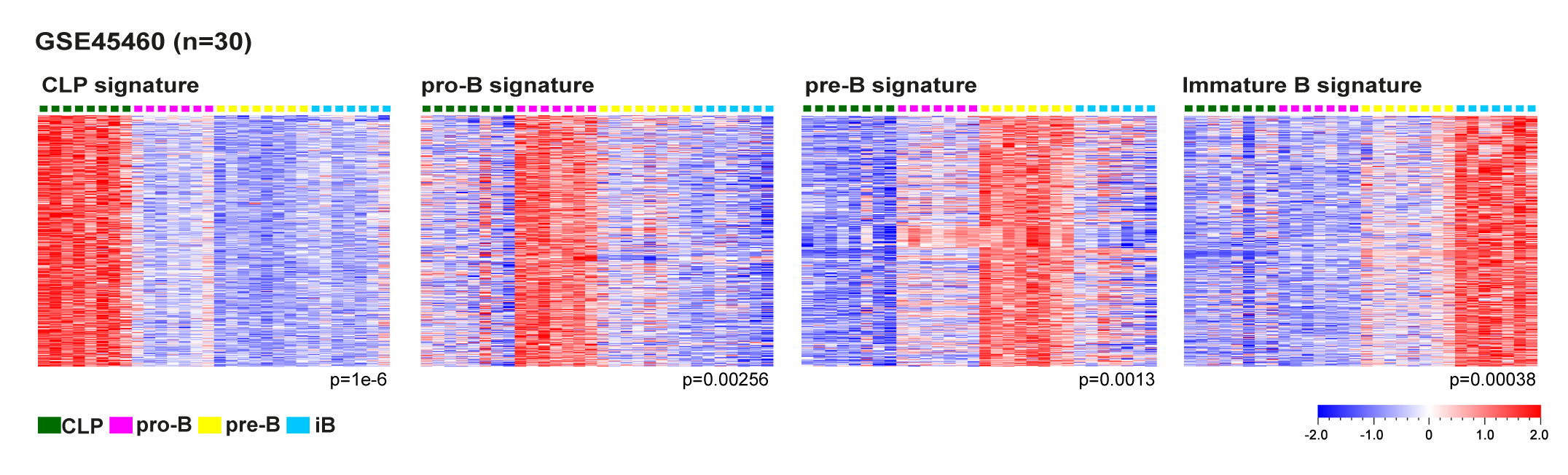

Supplement: S2 Fig — The top 400 genes highly expressed in different developmental stages (CLP, pro-B, pre-B and iB signatures) were identified using supervised comparison in the data set including samples from healthy donors (GSE45460). iB, immature B. (TIF) [file pone.0162638.s002.tif]

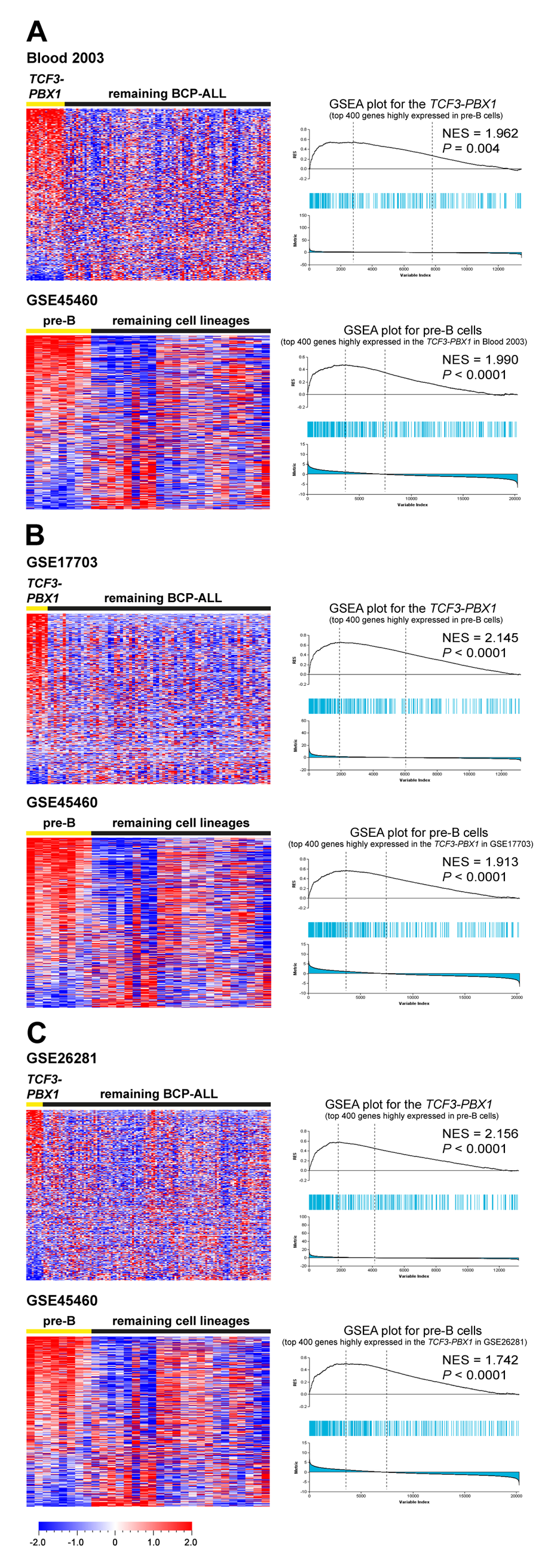

Supplement: S3 Fig — (A-C) Upper: Heat map and enrichment plots show the pre-B signature in the TCF3-PBX1 and the remaining BCP-ALL in the indicated data sets. Lower: Heat map and enrichment plots show the TCF3-PBX1 signature in pre-B cells and the remaining developmental stages. (TIF) [file pone.0162638.s003.tif]

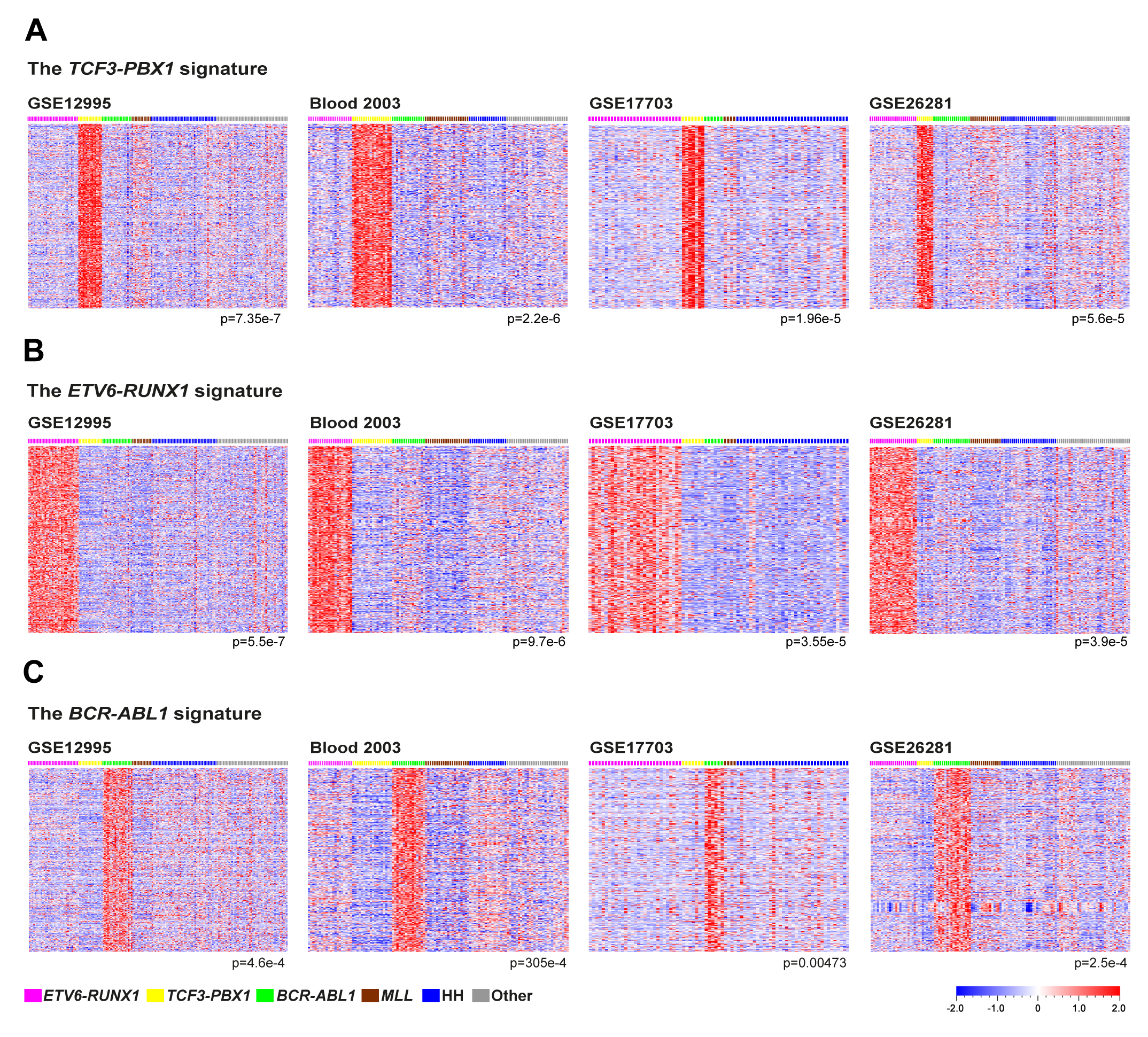

Supplement: S4 Fig — (A) The top 400 genes highly expressed in the TCF3-PBX1 BCP-ALL (TCF3-PBX1 signature) are identified using supervised comparison. (B) The top 400 genes highly expressed in the ETV6-RUNX1 BCP-ALL (ETV6-RUNX1 signature) are identified using supervised comparison. (C) The top 400 genes highly expressed in the BCR-ABL1 BCP-ALL (BCR-ABL1 signature) are identified using supervised comparison. (TIF) [file pone.0162638.s004.tif]

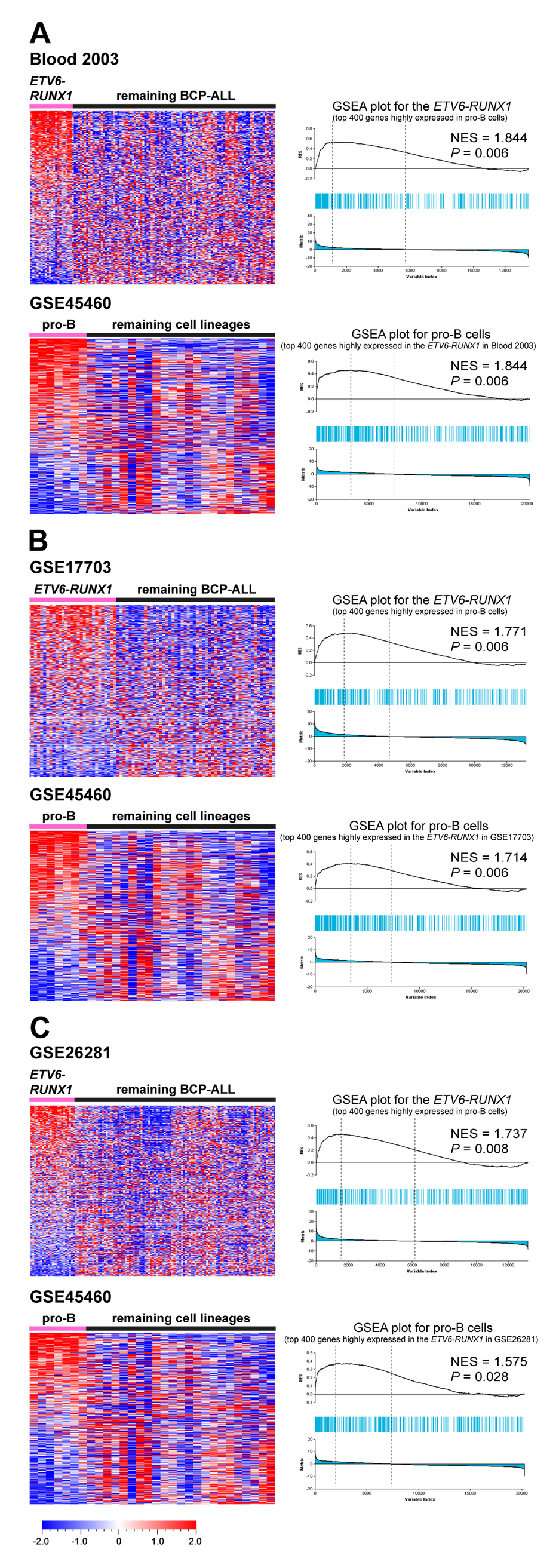

Supplement: S5 Fig — (A-C) Upper: Heat map and enrichment plots show the pro-B signature in the ETV6-RUNX1 and the remaining BCP-ALL in the indicated data sets. Lower: Heat map and enrichment plots show the ETV6-RUNX1 signature in pro-B cells and the remaining developmental stages. (TIF) [file pone.0162638.s005.tif]

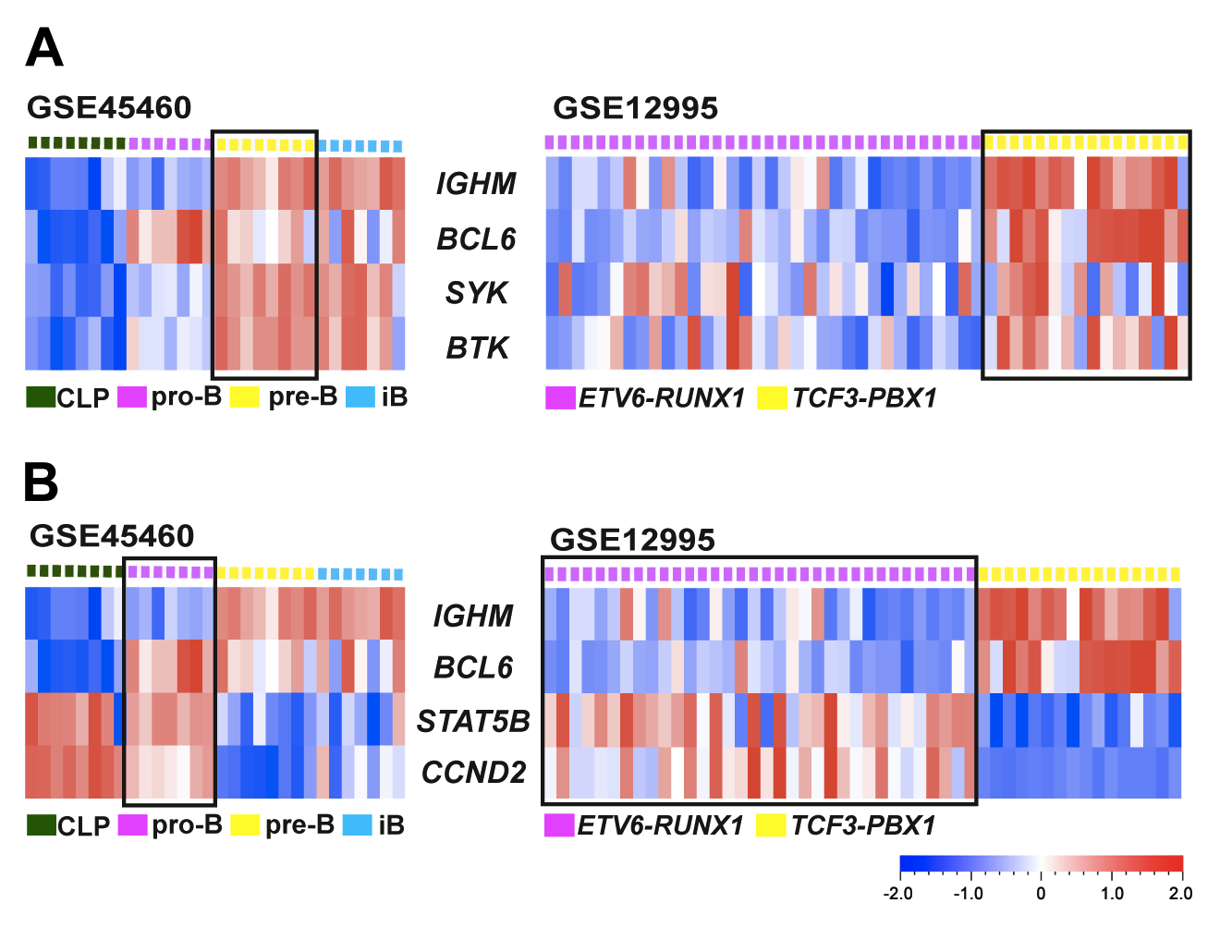

Supplement: S6 Fig — (A and B) Heat maps show the expression patterns of indicated molecules in normal B-cell (GSE45460) and in childhood BCP-ALL (GSE12995) data sets. (A) IGHM, BCL6, SYK and BTK expression in pre-B cells and TCF3-PBX1 (boxed). (B) IGHM, BCL6, STAT5B and CCND2 expression in pro-B cells and ETV6-RUNX1 (boxed). iB, immature B cells. (TIF) [file pone.0162638.s006.tif]
