# Supplementary material for: The Expression Pattern of the Pre-B Cell Receptor Components Correlates with Cellular Stage and Clinical Outcome in Acute Lymphoblastic Leukemia
Source: PLoS One. 2016 Sep 9;11(9):e0162638. doi: 10.1371/journal.pone.0162638 (PMC5017602; doi:10.1371/journal.pone.0162638)
Supplement: S1 Table — (DOCX) [file pone.0162638.s011.docx]

|  | **Protein (Flow Cytometry)** | | | | | | | |  | **Copy Number Variation (SNP)^@^** | | | | |
| --- | --- | --- | --- | --- | --- | --- | --- | --- | --- | --- | --- | --- | --- | --- |
| **Pat #** | **CD19^+^- blasts, %** | **CD10** | **CD34** | **TDT ic** | **IGHM ic** | **VPREB1 ic, %** | **IGLL1 ic, %** | **CD79A ic** | **Genetic alterations** | ***IGHM***  **(14q32.33)** | ***VPREB1***  **(22q11.22)** | ***IGLL1***  **(22q11.23)** | ***CD79A***  **(19q13.2)** | ***CD79B***  **(17q23.3)** |
| 1 | 45 | + | - | + | + | +**^*^** | 39 | + | other | Amp | - | - | - | - |
| 3 | 88 | +B | + | + | +D | 69 | nd | + | HH | Amp | - | - | - | - |
| 4 | 91 | +B | S52% | + | - | 42 | nd | + | HH | Amp | - | - | - | Amp |
| 6 | 95 | +B | + | +D | - | 82 | nd | + | *ETV6*-*RUNX1* | Amp | Del | - | - | - |
| 7 | 89 | +B | +H | +H | S20% | <1 | nd | + | Other, DS | - | Del | - | - | Amp |
| 8 | 85 | +B | S28% | + | + | 71 | 71 | + | HH | - | Amp | - | - | Amp |
| 9 | 81 | + | + | +D | - | 55 | 15 | + | HH | Amp | - | - | - | Amp |
| 10 | 90 | +B | +H | + | + | 74 | 57 | +B | HH, *BCR*-*ABL1* | Amp | Amp | Del | - | - |
| 11 | 94 | + | + | + | +D | 91 | 88 | + | other | Amp | Del | - | - | - |
| 12 | 93 | + | - | + | + | 86 | 74 | + | *TCF3*-*PBX1* | Amp | Amp | - | - | - |
| 13 | 62 | +D | +DH | nd | nd | <1 | 50 | + | *ETV6*-*RUNX1* | - | Double del | - | - | - |
| 14 | 91 | - | S29% | - | - | 38 | 15 | nd | *MLL* | - | - | - | - | - |
| 15 | 90 | + | + | + | - | <1 | <1 | + | HH | Amp | - | - | - | Amp |
| 16 | 85 | +H | + | + | - | 10 | <1 | + | other | Amp | Amp | Amp | - | - |
| 17 | 85 | +B | + | + | + | 16 | <1 | + | HH | Amp | - | - | - | Amp |
| 18 | 85 | +B | S36% | + | - | 20 | 10 | + | *ETV6*-*RUNX1* | - | - | - | - | - |
| 19 | 78 | + | + | + | + | 66 | 57 | + | HH | Amp | - | - | - | - |
| 20 | 85 | +B | - | + | +D | 36 | 20 | + | other | Amp | - | - | - | - |
| 21 | 75 | +B | + | + | S85% | 25 | 25 | + | HH | Amp | - | - | - | Amp |
| 22 | 78 | + | + | + | - | <1 | <1 | + | HH | Amp | - | - | - | Amp |
| 23 | 32 | +B | - | + | - | nd | nd | + | HH | Amp | - | - | - | - |
| 24 | 90 | + | +H | + | + | nd | nd | + | HH | Amp | - | - | - | Amp |
| 25 | 80 | +B | +H | + | - | nd | nd | +H | *ETV6*-*RUNX1* | nd | nd | nd | nd | nd |
| 27 | 81 | +B | + | +D | +DH | nd | nd | +DH | *ETV6*-*RUNX1* | Amp | - | - | - | - |

B, bright; CD79A, Igα; CD79B, Igβ; D, dim; DS, Down syndrome; H, Heterogeneous; HH, High Hyperdiploid; S, subset; nd, not determined

*, surface staining; **^@^ -,** Amplifications or Deletions were not detected
