# Supplementary material for: The Expression Pattern of the Pre-B Cell Receptor Components Correlates with Cellular Stage and Clinical Outcome in Acute Lymphoblastic Leukemia
Source: PLoS One. 2016 Sep 9;11(9):e0162638. doi: 10.1371/journal.pone.0162638 (PMC5017602; doi:10.1371/journal.pone.0162638)
Supplement: S2 Table — (DOCX) [file pone.0162638.s012.docx]

| **Subtype** | ***IGHM***  **(14q32.33)** | | ***VPREB1***  **(22q11.22)** | | ***IGLL1***  **(22q11.23)** | | ***CD79A***  **(19q13.2)** | | ***CD79B***  **(17q23.3)** | |
| --- | --- | --- | --- | --- | --- | --- | --- | --- | --- | --- |
|  | **Amp** | **Del** | **Amp** | **Del** | **Amp** | **Del** | **Amp** | **Del** | **Amp** | **Del** |
| ***ETV6-RUNX1*** | 43%  (12/28) | 18%  (5/28) | 11%  (3/28) | 29%  (8/28) | 14%  (4/28) | 4%  (1/28) | 4%  (1/28) | 0%  (0/28) | 4%  (1/28) | 4%  (1/28) |
| ***TCF3-PBX1*** | 100%  (4/4) | 0%  (0/4) | 25%  (1/4) | 0%  (0/4) | 0%  (0/4) | 0%  (0/4) | 0%  (0/4) | 0%  (0/4) | 0%  (0/4) | 0%  (0/4) |
| ***BCR-ABL1*** | 67%  (2/3) | 0%  (0/3) | 33%  (1/3) | 33%  (1/3) | 0%  (0/3) | 33%  (1/3) | 0%  (0/3) | 0%  (0/3) | 0%  (0/3) | 0%  (0/3) |
| ***MLL*** | 56%  (5/9) | 0%  (0/9) | 0%  (0/9) | 11%  (1/9) | 0%  (0/9) | 0%  (0/9) | 0%  (0/9) | 0%  (0/9) | 0%  (0/9) | 0%  (0/9) |
| **Other** | 48%  (14/29) | 7%  (2/29) | 10%  (3/29) | 24%  (7/29) | 14%  (4/29) | 0%  (0/29) | 0%  (0/29) | 0%  (0/29) | 7%  (2/29) | 0%  (0/29) |
| **Total** | **51%**  **(37/73)** | **10%**  **(7/73)** | **11%**  **(8/73)** | **23%**  **(17/73)** | **11%**  **(8/73)** | **3%**  **(2/73)** | **1%**  **(1/73)** | **0%**  **(0/73)** | **4%**  **(3/73)** | **1%**  **(1/73)** |
| **HH** | 92%  (46/50) | 0%  (0/50) | 8%  (4/50) | 4%  (2/50) | 4%  (2/50) | 2%  (1/50) | 2%  (1/50) | 6%  (3/50) | 70%  (35/50) | 0%  (0/50) |
| **Total** | **67%**  **(83/123)** | **6%**  **(7/123)** | **10%**  **(12/123)** | **15%**  **(19/123)** | **8%**  **(10/123)** | **2%**  **(3/123)** | **2%**  **(2/123)** | **2%**  **(3/123)** | **31%**  **(38/123)** | **1%**  **(1/123)** |

HH, High hyperdiploid
