# Supplementary material for: The Expression Pattern of the Pre-B Cell Receptor Components Correlates with Cellular Stage and Clinical Outcome in Acute Lymphoblastic Leukemia
Source: PLoS One. 2016 Sep 9;11(9):e0162638. doi: 10.1371/journal.pone.0162638 (PMC5017602; doi:10.1371/journal.pone.0162638)
Supplement: S3 Table — The top 400 genes highly expressed at each of the four B cell developmental stages in data set DS5 were projected into data sets DS1-4 (Healthy -> Leukemia) and vice versa (Leukemia -> Healthy). Then gene set enrichment analyses were performed to determine the similarity of molecular signatures between BCP-ALL and normal B cells. Only significant p-values (>0.05) are shown. (DOCX) [file pone.0162638.s013.docx]

|  |  | **Healthy -> Leukemia** | | | |  | **Leukemia -> Healthy** | | | |
| --- | --- | --- | --- | --- | --- | --- | --- | --- | --- | --- |
| **Subtype** | **ALL data sets** | **Developmental stage, DS5 (n=30)** | | | |  | **Developmental stage, DS5 (n=30)** | | | |
|  |  | **CLPs (n=8)** | **pro-B (n=7)** | **pre-B (n=8)** | **iB (n=7)** |  | **CLPs (n=8)** | **pro-B (n=7)** | **pre-B (n=8)** | **iB (n=7)** |
| ***ETV6-***  ***RUNX1*** | **DS1 (n=34)** |  | **0.004** |  |  |  |  | **0.004** |  |  |
|  | **DS2 (n=29)** |  | **0.006** |  |  |  |  | **0.006** |  |  |
|  | **DS3 (n=20)** |  | **0.006** |  |  |  |  | **0.002** |  |  |
|  | **DS4 (n=23)** |  | **0.008** |  |  |  |  | **0.028** |  |  |
| ***TCF3-***  ***PBX1*** | **DS1 (n=16)** |  |  | **< 0.001** |  |  |  |  | **< 0.001** |  |
|  | **DS2 (n=7)** |  |  | **< 0.001** |  |  |  |  | **< 0.001** |  |
|  | **DS3 (n=18)** |  |  | **0.004** |  |  |  |  | **< 0.001** |  |
|  | **DS4 (n=8)** |  |  | **< 0.001** |  |  |  |  | **< 0.001** |  |
| ***BCR-***  ***ABL1*** | **DS1 (n=20)** |  | **0.011** |  |  |  |  |  |  |  |
|  | **DS2 (n=6)** |  |  |  |  |  | **0.019** |  |  |  |
|  | **DS3 (n=15)** | **0.033** | **0.004** |  |  |  |  |  |  |  |
|  | **DS4 (n=18)** |  | **0.035** |  |  |  |  |  |  |  |
| ***MLL*** | **DS1 (n=13)** |  |  |  |  |  |  |  |  |  |
|  | **DS2 (n=4)** |  |  |  |  |  |  |  |  |  |
|  | **DS3 (n=20)** |  |  |  |  |  |  |  |  |  |
|  | **DS4 (n=15)** |  |  |  |  |  |  |  |  |  |
| **HH** | **DS1 (n=44)** |  |  |  |  |  |  |  |  |  |
|  | **DS2 (n=35)** |  |  |  |  |  |  |  |  |  |
|  | **DS3 (n=17)** |  |  |  |  |  |  |  |  |  |
|  | **DS4 (n=27)** |  |  |  |  |  |  |  |  |  |
| **Other** | **DS1 (n=48)** |  |  |  |  |  |  |  |  |  |
|  | **DS2 (n=0)** |  |  |  |  |  |  |  |  |  |
|  | **DS3 (n=28)** |  |  |  |  |  |  |  |  |  |
|  | **DS4 (n=36)** |  |  |  |  |  |  |  |  |  |

iB, Immature B cells; DS, data set. DS1-4 (BCP-ALL), DS5 (healthy). DS1: GSE12995; DS2: GSE17703; DS3: Blood 2003; DS4: GSE26281; DS5: GSE45460.
